# Influencing Aquatic Invasive Species Prevention Behaviors: An Exploration and Experiment with Augmented Reality

**DOI:** 10.1007/s00267-025-02283-2

**Published:** 2025-10-01

**Authors:** Ingrid E. Schneider, Megan M. Weber, Anupa Khadka, Brock Bahlmann

**Affiliations:** 1 Department of Forest Resources, University of Minnesota, St. Paul, MN USA; 2 Andover Regional Office, University of Minnesota Extension, Andover, MN USA; 3 Minnesota Aquatic Invasive Species Research Center, St. Paul, MN USA

**Keywords:** Prevention, Mitigation, Information, Engagement

## Abstract

Despite significant resource allocations to thwart invasive species introduction and spread, they remain a significant issue for environments and economies alike. Aquatic invasive species (AIS) are of particular interest given the value of water within and across societies. AIS prevention activities typically include outreach and education campaigns reliant on static signage which leave opportunities for greater engagement. Addressing a primary pathway for AIS spread, this project compared the impact of static and dynamic message mediums on boating anglers’ intentions to perform AIS prevention behaviors and assessed the impact of various variables on the likelihood to perform AIS prevention behaviors. Survey results of boating anglers in one U.S. Midwestern state revealed very high self-reported intentions for prevention behaviors and no significant differences across message mediums (sign, augmented reality or simple AIS definitions). Therefore, judicious evaluation of decisions about management communication efforts remains essential across new and existing mediums.

## Introduction

Environmental managers face a number of challenges, invasive species among them (Colberg et al. 2024). Invasive species are a major driver of biodiversity loss, ecosystem disruption, and ecosystem service degradation (Clavero et al. 2009; Pyšek and Richardson 2010; Nentwig 2007). Despite significant resource allocations to thwart their introduction and spread, invasive species remain a significant issue for environments and economies alike. Between 1980 and 2019, the global economic burden of invasive species reached $1.2 trillion: this 702% rise was faster and costlier than many natural hazards (Turbelin et al. 2023). Preventing the transport of invasive species is paramount (Vander Zanden and Olden 2008) and cost-effective compared to remediation (Ahmed et al. 2022; Lovell et al. 2006).

Aquatic invasive species (AIS) are of particular interest given the value of water within and across societies (Buczkó et al. (2022); Singh 2021; Pradhananga et al. 2015), the significance of the water-based recreation industry (Outdoor Participation Trends Report 2023), and communities dependent on that industry. Water is valued for a variety of reasons, with some variation across demographic markers (Pradhananga et al. 2015). The recreational value of water is both social and economic. In 2023, fishing alone contributed $35.8 billion in U.S. consumer spending (Outdoor Participation Trends Report 2023). While valuable, water-based recreational activities also serve as an AIS pathway, especially boating anglers due to their frequent movement (Harrower et al. 2018; Morreale et al. 2023; Minnesota Department of Natural Resources (2021): Kinsley et al. 2022; Ukić Boljat et al. 2021; Ielmini and Sankaran 2021).

Prevention activities typically include outreach and education. Informational efforts have proliferated as they are relatively low cost and indirect approaches that retain recreationist freedoms (Haley et al. 2023; Manning et al. 2017; Seekamp et al. 2016; Witzling et al. 2015). Effective outreach and education can significantly enhance pro-environmental knowledge, attitudes, and intentions (Ardoin et al. 2015; Kidd et al. 2019; Newsome et al. 2013). While laudable, the efficacy and ongoing effectiveness of informational efforts deserves attention to optimize resource use and effectiveness (Haley et al. 2023; Turbelin et al. 2022; Weingart et al. 2021). A review of invasive species awareness studies found just a few of the twenty-four evaluated were successful (Haley et al. 2023). Within a single Midwestern U.S. state, AIS prevention behavior performance did not significantly differ among regions despite significant public outreach investments (Cole et al. 2016). Thus, education campaign effectiveness remains unclear as does detailed information on angler AIS perceptions and actions (Moore et al. 2024).

Onsite informational efforts rely heavily on signage. Like other options within the information management suite, signs are appealing due to their relatively low investment, simplicity, and angler preferences for them versus other communication mediums (Selvaag et al. 2022; Nathan et al. 2014; Lindgren 2006; Witzling et al. 2015; Witzling et al. 2016). However, their effectiveness is questionable for at least two reasons. First, within the AIS realm, research reveals signs are not frequently read or are, at best, glanced at (Fortin Consulting 2020; Hennepin County 2018; Bolton and Menk 2023; Authors, in revision). Second, the static nature of most signs reduces their effectiveness (Ahn et al. 2014; Alyahya and McLean 2021; Cai 2013; Marto et al. 2019; Petty and Cacioppo 1986). While research examines AIS message content (Fitzgerald and Donnelly 2020; Golebie and van Riper 2023; Wallen and Kyle 2018; Kolandai-Matchett and Armoudian 2020; Shaw et al. 2021), message medium research remains limited.

In the 21st century, communication is increasingly dynamic as advanced communication technologies (ACT) offer a breadth of opportunities beyond static signage. Understanding the efficacy of dynamic messaging mediums to influence AIS preventative behaviors is nascent but necessary (Schneider et al. 2023; Winter et al. 2020). Since the turn of the 21st century, research has revealed that pictures or other static visuals of environmental scenes were deemed less acceptable than dynamic ones (Heft and Nasar 2000). Beyond dynamic images, augmented reality (AR) offers greater engagement opportunities as it “augments” real environments by means of virtual objects (Milgram et al. 1994) with audio and visual attributes (Smith 2015). In vision-based AR, users point a camera at an item that has a QR or similar type of code and this code connects them to virtual images in their real-world contexts through enhanced computer-generated sensory inputs (Chen and Tsai 2012; Wu et al. 2013). In 2016, the “Pokemon Go” AR game swept the world. Depending on the application, users can interact with the virtual objects while still visualizing the world around them thus enhancing learning and desired attitude changes (Cai 2013; Howard 2017). For example, AR exposure elevated overall tourist experiences (Harley et al. 2020) and produced more positive attitudes about a destination (Chung (2018)). While AR has been employed to create AIS-related games (Howard 2017), it has neither been applied to AIS-prevention behaviors nor evaluated for effectiveness.

Beyond the message medium, consideration to the message recipient is warranted as persuasion theory suggests personal factors influence AIS behavioral intentions. Specifically, the elaboration likelihood model (ELM) suggests issue relevance and prior knowledge (Petty and Cacioppo 1986) are key influences, while other theories suggest environmental values (Golebie and van Riper 2023; Pradhananga et al. 2015; Johnson et al. 2001) and efficacy (Hutchins et al. 2023; Golebie and van Riper 2023) also play a role.

Thus, in response to calls for expanded research on AIS prevention message effectiveness (Haley et al. 2023; Kolandai-Matchett and Armoudian 2020) and the opportunity to include ACT, this project addressed if and how increasingly engaging mediums impacted boating angler intentions to perform AIS preventative behaviors. We sought to (1) compare the impact of static and dynamic message mediums on boating anglers’ intentions to perform AIS prevention behaviors and (2) assess the importance of message medium and personal characteristics on the likelihood to perform AIS prevention behaviors with predictive modeling. We hypothesized that as the message medium increased in engagement, self-reported likelihood to perform AIS prevention behaviors would increase. Based on past research and theory, we also hypothesized that personal variables would increase likelihood to perform AIS prevention behaviors.

## Literature Review

Since the 1980s, persuasion models have suggested highly engaging information is more likely to impact and alter attitudes and behaviors as it enables the recipient to thoroughly process and elaborate on the content (Petty and Cacioppo 1986). The ELM, a dual-process theory of persuasion, describes how attitudes are formed and changed based on a person’s involvement and motivation (Petty et al. 1992). The two processes are central and peripheral. While the peripheral route requires little processing effort and typically induces only short-term changes, the central route requires increased cognitive effort and relies primarily on the message content rather than external contextual cues (Petty and Cacioppo 1986; Petty et al. 1992). Messages processed through the central route typically result in more enduring, predictive, and preferred attitudes (Petty et al. 1992). According to the ELM, individuals differ in their capacity and motivation to elaborate on an argument, which influences how the information will shape their attitudes (Bhattacherjee and Sanford 2006).

Elaboration depends on engagement and interactivity. Engagement is typically defined as attending to the content (Shin 2019; Bitgood 2009) whereas interactivity is the “extent to which users can participate in modifying the form and content of a mediated environment in real-time” (Steuer 1992 p. 84) and positively related to elaboration (Oh and Sundar 2015). Elaboration has been deemed “integral” for influencing AIS beliefs and a key goal for prevention behavior messaging (Golebie and van Riper 2023).

Various communication interventions, including AR, can employ interactive, engaging “rich-media” environments (Daft and Lengel 1986). The real-time interactivity (Azuma 1997) and real-world enhancement can create engaging experiences (Du Toit 2018; Yovcheva et al. 2013). For example, Mauroner et al. (2016) found AR interactions significantly enhanced brand recall and more favorable attitudes toward the product. Similarly, Mirauda et al. (2017) demonstrated a mobile AR experience significantly enhanced both the efficiency and accuracy of water monitoring activities. In a terrestrial invasive species application (Schneider et al. 2023), AR experiences compared to static text experiences significantly impacted forest visitors’ landscape preferences, but not their intentions to leave impacted areas.

The combination of AR and individual factors influencing AIS-prevention behaviors is unexplored but deserves attention. Familiarity has been studied in outdoor recreation management, primarily as experience in an area or with an issue (Manning 2022; Moscardo and Hughes 2023). In terms of AIS, familiarity and experience with AIS impacted the perceptions of problem severity and prevention behavioral intentions (Moore et al. 2024) as well as likelihood to pay for AIS prevention (Levers and Pradhananga 2021). Similarly, past behavior has reliably predicted behavioral intentions (Ajzen 1991; Ouellette and Wood 1998; Albarracin and Wyer 2000; Kemp et al. 2017). The repetition and success of past behavior influence their continued performance. For example, past behavior impacted intentions for compliant fishing behaviors among Chileans (Vallejos et al. 2023). Further, since the early 2000s, research has demonstrated a positive relationship between familiarity, behavioral intentions, and self-efficacy (Morris, Dinther (2011); Moore et al. 2024).

Efficacy takes two forms: self and response (Rogers 1975). Self-efficacy is an individual’s belief that they will be able to accomplish a specific task (Bandura 1977), highly dependent on positive past experiences as well as expectations of how well one will perform a certain task. Since its introduction, self-efficacy has been researched in a variety of contexts from climate change to medical treatments (Krantz and Monroe 2016; Myhre et al. 2020; Pradhananga and Davenport 2022; Snyder and Swann 1976) and terrestrial invasive species management (Clarke et al. 2021). Recognized in the 2020 s with regard to AIS and boating anglers, “the role of self-efficacy …may be especially important given that anglers may be aware of the steps necessary to prevent the spread of AIS but still not take them” (Hutchins et al. 2023 pg. 15). Response efficacy refers to a belief that one’s actions will be effective (Bradley et al. 2020). In contrast to self-efficacy that focuses on an ability to perform, response efficacy addresses the perception of risk as a motivator to take action and the judgment of the probability and severity of current or future harm (Bradley et al. 2020). The statistical significance of response- and self-efficacy as AIS prevention behavior predictors has varied in the few studies including them. Self-efficacy was the strongest of three predictor variables for boater behavioral intentions to minimize AIS spread (Moore et al. 2024) and twice as important as response efficacy when predicting AIS prevention behaviors among wading or shore anglers (Hutchins et al. 2023). Among other boaters, each were significant predictors of AIS prevention behaviors but predictive capability varied by activity: self-efficacy was the strongest predictor of boaters while response-efficacy was the strongest predictor for anglers cleaning fishing equipment (Golebie et al. 2023).

In addition, an enduring line of research demonstrates value orientations influence certain pro-environmental behaviors (Stern 2000; Nordlund and Garvill 2002; Oreg and Katz-Gerro 2006). Values are inclusive concepts of desirable behaviors that go beyond specific situations, guide the selection or evaluation of actions and events, and are prioritized by their relative importance (Schwartz and Bilsky 1990). Biocentric and anthropocentric values represent distinct paradigms for interacting with nature. While biocentric perspective views nature as intrinsically valuable, irrespective of its utility to humans. In contrast, anthropocentric values prioritizes human needs, interests, and well-being at the center of moral consideration (Abrams et al. 2005; De Groot and Steg 2008; Vaske and Donnelly 1999). Values have been explored since the mid 2000s in relation to AIS prevention behaviors. As in other realms (Schultz et al. 2005), biocentric values have predicted AIS concern which, in turn, predicted angler intentions to perform prevention behaviors (Pradhananga et al. 2015), positively related to support of aquatic ecosystem protection (McCumber, Stedman (2023), and predicted AIS risk perceptions more than other orientations (Golebie et al. 2023). In addition, message frames focused on biospheric and altruistic values significantly enhanced their persuasive impact among recreationists in Illinois, fostering stronger beliefs in their ability to take action against AIS spread (Golebie et al. 2023).

In response to the call for critical evaluation of campaigns (Haley et al. 2023; Smith et al. 2020) and based on persuasion theory and previous work, we included both personal and message factors to assess (1) the impact of static and more engaging message mediums on boating anglers’ intentions to perform AIS preventative behaviors, and (2) the importance of personal characteristics on intentions to perform AIS preventative behavior. We hypothesized the AR messages would increase behavioral intentions to perform prevention behaviors as would AIS familiarity, biospheric values, and efficacy.

## Methodology

A multi-method approach addressed the influence of personal and message medium variables on the likelihood of boating anglers taking AIS prevention behaviors. Throughout a 12-month period we observed, interviewed, and surveyed boating anglers to understand their behaviors and glean insights on how to positively influence their behavioral intentions. In this paper, we focus on the survey outcomes.

### Study Site and Sample Selection

Onsite questionnaires were completed at a sports show in the upper Midwest of the United States where boating anglers were a primary audience among the estimated 20,000 attendees. At the sportshow we secured a booth where we had trained staff, colorful signs, and the promise of a brand-name lure that enticed attendees to participate in the project. Interested attendees were screened to ensure they were adults (18 years or older), did not have formal training in AIS inspection or removal, and had fished from a boat in the last 12 months. If they qualified, they were invited to complete a questionnaire that took an average of 12 min to complete on a provided Apple iPad.

The sports show was hosted in what is now known as the state of Minnesota in the United States. Minnesota, located on Dakota and Anishinaabe lands, derives its name from the Dakota language, meaning “water that reflects the skies.” Minnesota residents highly value the abundant water resources (Davenport et al. 2024). AIS spread in Minnesota is particularly relevant with its more than 14,380 lakes and other water bodies as well as its connectivity to the Great Lakes, which contain more than 21% of the world’s surface freshwater (Downing 2021; Morreale et al. 2023).

### Questionnaire

The multi-section questionnaire was developed based on previous literature, observations, and interviews conducted the year prior. A pre-test indicated we needed to reduce the length and be more explicit about stopping the questionnaire to get the message medium treatment. Relevant variables for this analysis include AIS familiarity, perceived problem severity, past performance of preventative actions, likelihood of future prevention behavior intentions, values, and self- and response-efficacy. AIS familiarity and experience was measured summing three yes or no items assessing experience fishing at an area infested with AIS (lake most frequently fished, any lake fished, lake with visual impairment due to AIS). Similarly, severity was measured asking if and how much of a problem AIS were, from “not a problem at all” to “extreme problem” on a five-point scale. Following previous research (Pradhananga et al. 2015; Golebie et al. 2021b), eight past AIS prevention actions were measured on a seven-point scale from “never” to “always.” Value orientations were measured using 10 items, categorizing responses into anthropocentric (six items) and biocentric (four items) orientations, with a seven-point scale from “strongly disagree” to “strongly agree” (Pradhananga et al. 2015). Similarly, self- and response- efficacy scales were measured using seven items on a seven-point agreement scale with items such as “I am capable of performing the tasks required to remove AIS from my boat and equipment” and “If everyone remembered to perform the recommended behaviors, we could significantly lower the risk of AIS spread” (Golebie et al. 2023).

Respondents completed the same questions but were systematically assigned into different message medium treatments, with a random start each session: (1) control with text AIS definition, (2) control with AR AIS definition enhanced with videos and voiceover, (3) AR AIS prevention messaging focused on cleaning and draining behaviors with videos and voiceover, or (4) static sign AIS prevention messaging about cleaning and draining behaviors with photos and text. Immediately following their informational treatment, respondents were asked about their likelihood of performing AIS prevention behaviors related to draining or cleaning. Each behavior set had four items measured on a scale from “very unlikely” to “very likely.” While self-reported behavior has its challenges and there is a gap between reported and actual behavior, it informs the action-behavior gap levels and is also an effective way to gain insight for a number of people.

The message medium treatments were presented in the early part of the questionnaire. The control-text treatment was simply a statement about AIS included in the questionnaire. The control-AR treatment was this same AIS statement shared through an AR experience with interactive video, voice and ambient sounds. Sign and AR prevention behavior message treatments targeted cleaning behaviors and draining behaviors separately using the same words but varied levels of interactivity. The sign treatments shared photos and text about draining or cleaning boats and equipment to comply with state law and preferred behaviors. Respondents assigned to this treatment were directed to “see the staff” who then presented a 8 × 14 laminated sign about cleaning or draining for the respondent to read from a tabletop holder. Respondents could take as much or little time with the “sign” before returning to the questionnaire to respond to the behavioral intention questions about the topic and then were directed to read the other sign (cleaning or draining) and proceed with the intention questions. Respondents assigned to the AR experiences were also directed to “see the staff” who explained the respondent would be having an AR experience and offered headphones to minimize distractions, directed them to scan a code with the supplied Apple iPad, and follow the instructions on the iPad screen. In each interactive AR scene, respondents heard an introductory statement and were directed through three videos with touch icons during which they could move the tablet around to fully view the experience. The control-AR treatment was about 40 s and the draining and cleaning AR messages were ~50 s each (See Appendix/Supplemental material for sign-message and AR codes).

### Analysis

We used the Statistical Package for Social Sciences (SPSS version 28.0) to clean and analyze data. Prior to comparing or predicting behavioral intentions, we assessed missing data patterns, variable normality and relationships. No missing data patterns emerged. The draining and cleaning intention variables were not normally distributed per Kolmogorov-Smirnov tests (all items significant at 0.001). Predictor variables were normally distributed with neither collinearity nor singularity present.

We created scales for the cleaning and draining scales as well as their predictor variables. We used iterative factor analysis with oblimin rotation as the scale items were hypothesized to be related. Final scales included items with loadings of 0.6 or higher and reliability values of 0.70 or higher, using both Cronbach’s alpha and McDonald’s omega (Bagozzi and Yi 1988; Cortina 1993).

As the likelihood to perform AIS prevention behavior items were not normally distributed, we used the Kruskal Wallis test to assess any differences in intention by message medium among the boaters who moved their boats more than one time per season (*n* = 245; Witzling et al. 2015). To predict behavioral intentions for draining and cleaning intentions, we used general linear regression models with all variables entered simultaneously.

## Results

The selected questionnaire respondents were predominantly White (91.8%), male (81.1%) with an average age of 52.67 years, comparable to other research (*n* = 245; Minnesota Department of Natural Resources (2021). Nearly half the sample had fished for more than 40 years (46%), an average of 25.57 times in the last year, 23.88 times in Minnesota. While 23% had never heard of AR, most had at least heard of it (77%) with 8% who reported to know a lot about it. Nearly 45% had never used AR.

Prior to receiving treatments, the majority of respondents self-reported always cleaning their trailer (90.5%), their boat (85.7%), and trolling motor (88.8%) after fishing. Nearly 72% reported they always clean their fishing equipment, including rods and reels. Similarly, the majority reported always removing their drain plug before and after fishing (95%), draining water from every boat part (87.1%), and their motor (78.2%).

Post-treatment intentions to perform AIS prevention behaviors on the next outing were also very high (84% or higher). When comparing respondents across message mediums, their intentions to clean or drain boats and equipment did not significantly differ (Table 1).

**Table 1:** Kruskal-Wallis test comparing self-reported likelihood to perform aquatic invasive species prevention actions next time fishing by message medium among motorized boat anglers move two or more times in a season

		Treatment Mean Ranks				*H* value
Behaviors^a^		Control, Text (*n* = 51–55)	Control, Augmented reality (*n* = 59–62)	Static messages (*n* = 51–56)	Augmented reality messages (*n* = 43–47)	
Cleaning	Remove aquatic plants and animals from the boat	124.93	122.39	115.56	118.71	2.28
	Remove aquatic plants and animals from the trailer	107.22	118.40	115.80	116.91	4.53
	Remove aquatic plants and animals from fishing equipment	111.11	122.45	112.53	112.17	3.02
	Remove aquatic plants and animals from the trolling motor	109.23	115.55	114.22	117.57	1.93
Draining	Drain all water from all boat parts	112.32	124.19	119.69	114.49	4.28
	Drain all water from the motor	106.25	117.28	115.91	121.97	4.84
	Drain all water from any bait containers	112.76	120.58	114.21	115.78	1.22
	Remove drain plug	113.13	119.49	119.09	118.23	2.57

^a^Variable measured on a 7-point scale: 1 = Very unlikely and 7 = Very likely

### Factors Influencing Likelihood of Performing Preventive Behaviors

More than 75% of respondents indicated experience with AIS in the lakes they frequent, in other water bodies they visited and with visual impacts of AIS (Table 2). A majority perceived AIS as a “severe problem” in Minnesota (51.8%) while another 31% identified AIS as a “moderate problem”.

**Table 2:** Descriptive statistics (%) for experience with aquatic invasive species, means (SD in parentheses) and factor loadings for past behaviors, self-efficacy, response efficacy, and values reported by boating anglers in Minnesota

	% with experience	
Experience with AIS^a^ (*α* = 0.83, *Ω* = 0.83)		
AIS on the lake they fish frequently	77.7	
Visited waterbody with AIS	88.9	
Fished in waterbody visually impacted with AIS	77.9	

	Factor loading	Mean (SD)
Past cleaning behavior^b^ (*α* = 0.91, *Ω* = 0.91)		6.67 (0.74)
Remove aquatic plants and animals from the boat	0.81	6.71 (1.18)
Remove aquatic plants and animals from the trailer	0.87	6.70 (1.22)
Remove aquatic plants and animals from fishing equipment	0.71	6.63 (1.32)
Remove aquatic plants and animals from the trolling motor	0.87	6.62 (1.22)
Past draining behavior^c^ (*α* = 0.76, *Ω* = 0.73)		6.66 (0.70)
Remove drain plug	0.82	6.83 (0.86)
Drain all water from the motor	0.59	6.75 (0.89)
Drain all water from all boat parts	0.68	6.73 (1.08)
Drain all water from any bait containers	0.76	6.66 (1.09)
Self efficacy^d^ (*α* = 0.75, *Ω* = NA)		5.58 (1.96)
I understand what I need to do to prevent the spread of AIS.	0.82	5.61 (2.21)
I feel confident in performing procedures necessary to prevent the spread of AIS.	0.81	5.44 (2.35)
Response efficacy^e^ (*α* = 0.75, *Ω* = NA)		6.32 (1.26)
Cleaning my boat and equipment helps to prevent the spread of AIS.	0.82	6.45 (1.31)
If everyone remembered to perform the recommended behaviors, we could significantly lower the risk of AIS spread	0.86	6.21 (1.50)
Biocentric values^f^ (*α* = 0.63, *Ω* = 0.63)		5.30 (1.34)
Management should focus on doing what is best for nature instead what is best for people	0.80	5.60 (1.64)
Protecting the environment is more important than providing fishing opportunities	0.79	5.09 (1.78)
Fish have as much right to exist as people	0.66	4.98 (1.96)
Anthropocentric values^g^ (*α* = 0.81, *Ω* = 0.79)		2.29 (1.60)
Humans are meant to rule over the rest of nature	0.75	3.32 (2.10)
Humans have the right to change the natural environment to suit their needs	0.73	3.18 (2.06)
Fish are primarily valuable only as food to people	0.81	2.80 (2.00)
Fish are valuable only if people get to use them in some way	0.83	2.73 (1.97)

^a^Experience with AIS measured with nominal choices yes, no, unsure

^b^Past cleaning behavior measured on a seven-point scale, where 1 = never to 7 = always

^c^Past draining behavior measured on a seven-point scale, where 1 = never to 7 = always

^d^Self-efficacy measured using seven items on a seven-point scale, where 1=strongly disagree to 7 = strongly agree

^e^Response- efficacy scale measured using seven items on a seven-point scale, where 1 = strongly disagree to 7 = strongly agree

^f^Biocentric (four items) values measured on a seven-point scale, where 1 = strongly disagree to 7 = strongly agree

^g^Anthropocentric (six items) values measured on a seven-point scale, where 1 = strongly disagree to 7 = strongly agree

Select items were removed from each of the value and response-efficacy subscales to achieve higher reliability: biocentric (*α* = 0.63; *Ω* = 0.63), anthropocentric (*α* = 0.81; *Ω* = 0.79), self-efficacy (*α* = 0.75; *Ω* = NA), and response-efficacy (*α* = 0.75; *Ω* = NA). In terms of self-efficacy, respondents reported they had the capability to remove the AIS from boat and equipment (76.2%). However, they expressed low self-efficacy in their understanding of what they needed to do to prevent AIS spread (14.3%). Regarding response efficacy, respondents most strongly agreed that cleaning their boat and equipment helps to prevent the spread of AIS (74%) followed by the belief that if everyone followed the recommended behaviors, it would significantly reduce the risk of AIS spread (64.1%).

As the message medium did not influence intentions, we did not include it in the predictive models. Personal variables inconsistently predicted cleaning and draining intentions: significant but low variance was explained for draining behaviors only (*R*^2^ = 0.09; *p* < 0.05) where past draining behaviors (*β* = 0.324) and response-efficacy (*β* = 0.233) significantly predicted them Table 3.

**Table 3:** Results of multiple regressions predicting each type of aquatic invasive species prevention behavior

	Standardized β	
Independent variable	Cleaning intentions	Draining intentions
Problem severity	0.018	−0.028
Past draining behavior	0.138	0.324***
Past cleaning behavior	0.125	−0.085
Experience with AIS	0.065	0.152
Self-efficacy	0.015	0.036
Response efficacy	0.184	0.233**
Biocentric values	−0.014	0.062
Anthropocentric values	0.053	−0.025
Regression statistics		
Constant Adjusted R2	0.04	0.09
*F* value	1.89	3.08
df	8/157	8/158
*p*	0.06	<0.05

*** = significant at *p* < 0.001; **significant at *p* < 0.05

## Discussion

Onsite questionnaires explored boating anglers prevention behavior intentions and tested the impact of message medium on intentions to perform select behaviors. In contrast to hypotheses, (1) the more engaging medium neither consistently nor significantly impacted the intentions of boating anglers to perform prevention behaviors and (2) two of eight personal variables significantly predicted one of two suites of prevention behaviors. We discuss the results, implications, study limitations and opportunities for future research below.

Possible explanations for the lack of significant differences between static and AR messages relate to the research environment, the message medium, and a ceiling effect. Respondent concern about AIS and high self-reported performance of preventive behaviors mirrored past research (Golebie et al. 2021; Wallen and Kyle 2018; Moore et al. 2024; Witzling et al. 2016) and may also indicate a ceiling effect (Uttl 2005) where there is social desirability to report high prevention behaviors performance. Following our pre-test we did increase the scales from five to seven points but future research can consider even more scale points. In terms of the message medium, we employed best practices for the AR deployment, pretested the message medium, and provided directions to enhance and improve participant experiences. However, as the majority reported no prior AR knowledge, the novelty and perceived difficulty of use may have reduced the AR effectiveness. In addition, the AR experience included three messaging videos and perhaps a simpler version would be more effective for those with less AR experience (Saunders et al. 2019). Looking forward, as AR becomes more ubiquitous, the ease of use and experience complexity may present less of a concern. Finally, the study conditions may have diminished the message impacts by influencing engagement levels. While we took effort to diminish distractions by providing headphones to participants, other participants or sponsor announcements may have distracted them and limited message impacts and engagement (Petty and Cacioppo 1986; Slater and Steed 2000). As onsite messaging for outdoor consumers will invariably be in distracting environments, prudent consideration to the use and placement of AR messaging is essential. Integrating AR experiences into informational centers where distractions are lower, such as in a protected or shielded space, could increase the attention and engagement necessary for desired impacts.

Like past research, our results revealed a mixed influence of personal attributes on AIS prevention behavior intentions (Moore et al. 2024; Golebie et al. 2023; Kemp et al. 2017). Similar to Moore et al. (2024), past draining behavior predicted future draining behaviors. Therefore, enticing boating anglers to drain equipment and motors and instill it as a habit will serve AIS prevention efforts well. Particular attention to ensuring new and emerging anglers practice draining behaviors from the start can set a course to continue them. Past behavior has failed to predict future behavior if negative experiences occur or new challenges emerge (Albarracín and Wyer 2000). Given the surge in outdoor participants, including boating, exploring any new challenges at the launch such as boat density, impatient others or similar challenging situations is certainly worthwhile.

Respondents’ self and response efficacy did not consistently predict behaviors, in contrast to Golebie et al. (2023). One explanation may be the difference in outcome measures: Golebie et al. separated out fishing-specific equipment, including bait disposal, and we focused on draining behaviors. Consideration to fishing-specific equipment may be helpful in moving forward with messaging. Of course, as previously demonstrated, intended behaviors do not always match actual behaviors (Authors, in revision; Baumeister et al. 2007; Fortin 2020). Still, identifying them both is useful for AIS managers to understand any differences and then attempt to increase performance. For example, if boating anglers report they spend a certain amount of time cleaning and draining, but in fact, their time spent is much lower, that discordance could be used to change behavior.

In a related vein, population segments respond differently to environmental communications and thus require different intervention strategies (Connelly et al. 2016). Thus, while the AR messaging did not significantly differentiate behavioral intentions in this fairly experienced sample, with less-experienced anglers or specific segments, it may be more impactful and could be implemented. Further, despite the lack of significant influence on intentions, the AR technologies presented a novel, multi-generational engagement opportunity: all respondents were intrigued by the messaging medium and we informally observed those under 18 wistfully watching the AR experiment and even commenting on their adult companions as “lucky” to engage with the messaging. As AR becomes increasingly prevalent and commonplace, its effectiveness in improving angler intentions may improve.

Management implications include instilling good habits among new and emerging anglers, including response efficacy in communications, as well as prudent evaluation of existing and advanced campaigns and communication technologies like AR (Haley et al. 2023). As even a single boater or boat can spread invasive species, every single spread incident that is prevented is important. As past draining behavior predicted future draining intentions, instilling good habits among emerging and new anglers is essential as is encouraging experienced anglers to ensure they are performing the AIS prevention behaviors properly. Response-efficacy significantly impacted draining intentions and subsequently should be considered for inclusion in campaigns moving forward.

Attention to complementary interventions is also prudent as Angell et al. (2024) found a range of AIS-removal effectiveness among boaters, watercraft inspectors, and hot-water decontaminators. Beyond AR, perhaps it is possible to have boating anglers opt-in for messaging and, based on their activities or experience, receive targeted messaging for key issues at a location or places where AIS are missed (Johnson et al. 2001; Rothlisberger et al. 2010; Sharp et al. 2017).

### Limitations and Future Research

In this project, limitations include our sample and the measurement of likelihood rather than actual behavioral performance. First, sampling was conducted in a U.S. state where there have been heavy investments in AIS response, outreach, and education. Results may differ for regions where there has been less focus on AIS prevention campaigns and even different aquatic resources available. While the sample demographics reflected Minnesota’s overall boating angling population, increasingly anglers are more diverse racially and have a higher percentage female than our sample (Outdoor Industry Association 2023). Davenport et al. (2024) found differing water-value orientations among BIPOC residents, as such, future research can strive to target strata for the newer angler demographics. Second, our analysis focused on predicting likelihood to engage in behaviors that prevent the transport and spread of AIS (intentions) rather than tracking actual behaviors. Past research demonstrates that while behavioral intentions are an important construct to understand behaviors (Golebie et al. 2021a; Wallen and Kyle 2018; Moore et al. 2024; Witzling et al. 2016), self-report data may be skewed (Cimono and Stretcher 2018; Moore et al. 2024). Additional work by Authors (in revision) also suggests boating anglers perform prevention behaviors at rates lower than those self-reported in our questionnaire. Future research more directly connecting message medium to actual actions would be beneficial to fully understand the impact of message medium on AIS prevention such as with concurrent surveys and observations. As Smith et al. (2023) suggest, this angling AIS “situation is complex.”

Moving forward, future research can explore AR in a variety of formats and locations. One suggestion is to consider varying levels of AR complexity (Cipresso et al. 2018; Kim et al. 2024; Radu et al. 2023). Advanced communication technologies continue to evolve and therefore exploring the impact of simple to complex AR treatments may be useful. Comparing these types of complex messages with simpler and fewer messages could be advantageous to reduce development costs and participant time with the effectiveness of message interventions. In addition, comparing and testing message approaches could prove fruitful in the AR realm as Campbell et al. (2024) explored in video messaging and Schneider et al. (2023) explored in virtual reality messaging. As boating angling experience involves multiple trip phases from contemplation through travel and onsite, engaging boating anglers throughout the phases is advantageous to reinforce the message and prepare the anglers to perform the desired behaviors (Saunders et al. 2019). While onsite messaging is useful because messaging closer to the time to perform behaviors can be more influential (Saunders et al. 2019; Schwartz et al. 2018; Kolandai-Matchett and Armoudian 2020), repetition can also enhance preferred behavior performance. Beyond AR, perhaps it is possible to have recreationists opt-in for messaging and, based on their activities or experience, receive targeted messaging for key issues at a location or places where AIS are missed (Johnson et al. 2001; Rothlisberger et al. 2010; Sharp et al. 2017).

As managers continue the quest to prevent AIS spread, attention to evolving communications is essential to effectively address their audiences. While this initial foray into AR messaging revealed no significant impact on already high AIS prevention behavior intentions among the sample, other samples and settings may find greater success. Therefore, we recommend continued exploration of AR and other advanced communications among a variety of samples and settings.

## Supplementary information

Supplementary information

Supplementary information

Supplementary information

Supplementary information

Supplementary information

## Data Availability

The datasets generated during and/or analyzed as part of this study will be available in the Data Repository for University of Minnesota (DRUM).
