# Supplementary material for: Influencing Aquatic Invasive Species Prevention Behaviors: An Exploration and Experiment with Augmented Reality
Source: Environ Manage. 2025 Oct 1;75(12):3449–60. doi: 10.1007/s00267-025-02283-2 (PMC12575555; doi:10.1007/s00267-025-02283-2)
Supplement: Supplementary file 1 — Supplementary information [file 267_2025_2283_MOESM1_ESM.pdf]

Every surface you clean helps stop the spread of aquatic invasive species

You can protect our waters by taking the time to inspect and clean your boat, trailer, and equipment before you leave.

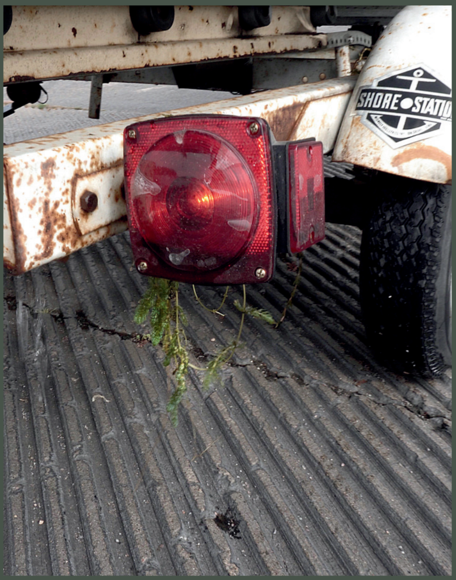

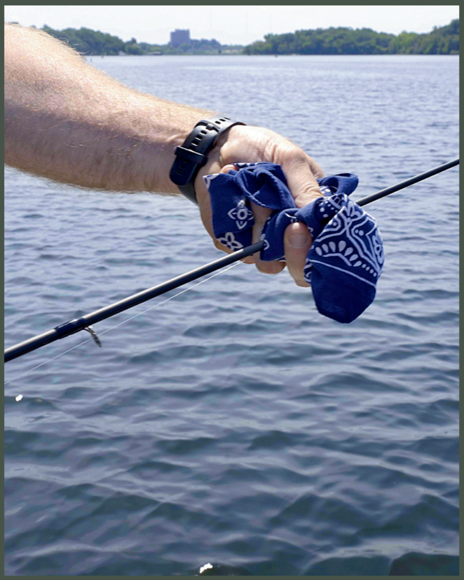

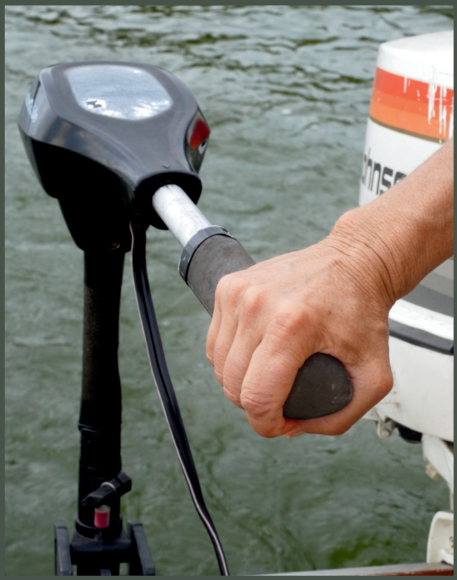

Trailers can be tricky!

Follow the law and avoid fines by **carefully cleaning surfaces** like cross beams, rollers, and wheel wells.

Your equipment matters too!

Even if they appear clean, it's important to **wipe lines and tackle**. Aquatic invasive species can be smaller than you think!

Have a trolling motor? Join other anglers in **checking the entire unit** to make sure invasive species are not catching a ride. Your actions ensure fish, friends and families can enjoy these waters for generations to come!
