# Supplementary material for: Influencing Aquatic Invasive Species Prevention Behaviors: An Exploration and Experiment with Augmented Reality
Source: Environ Manage. 2025 Oct 1;75(12):3449–60. doi: 10.1007/s00267-025-02283-2 (PMC12575555; doi:10.1007/s00267-025-02283-2)
Supplement: Supplementary file 2 — Supplementary information [file 267_2025_2283_MOESM2_ESM.pdf]

Leave the lake water behind

Invest your time to drain the water from your boat and gear before you leave to stop the spread of aquatic invasive species.

Join other anglers to drain every possible drop!

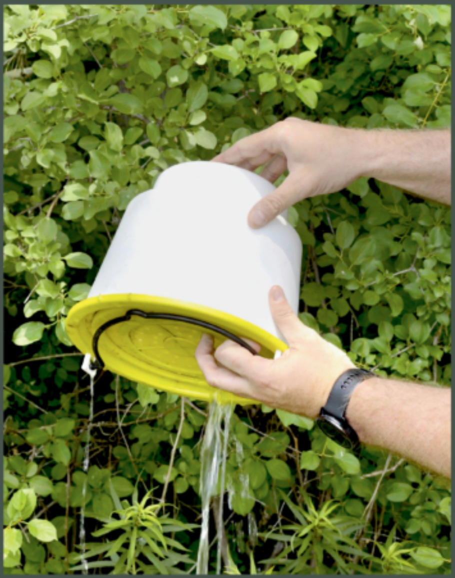

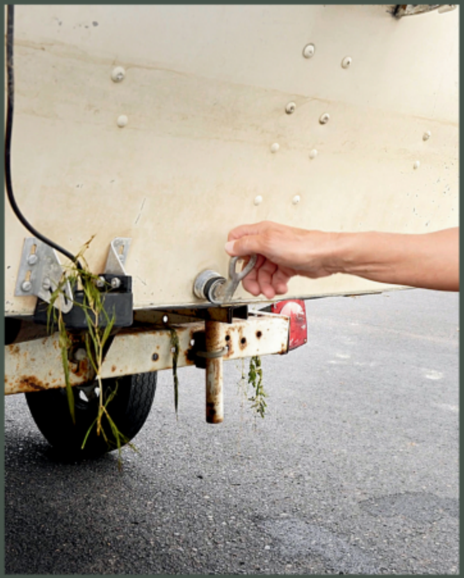

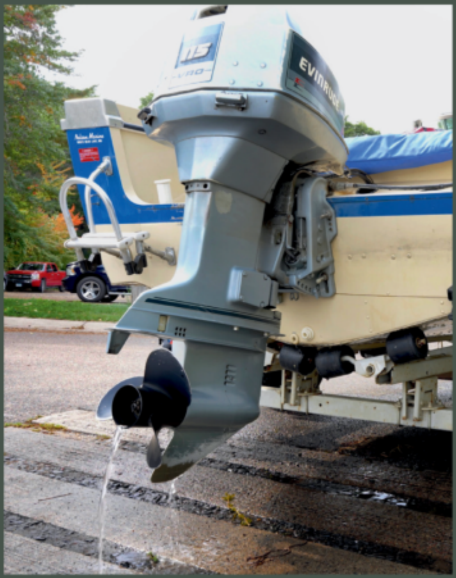

Using bait buckets and livewells? **Drain the lake water** to prevent the spread of invasive species and pack clean water to save your bait.

Pulling your drain plug protects our waters, preserves fishing experiences for everyone, and is the legal way to leave the launch.

You might be surprised by how much water your motor can hold. Start your cleaning routine by **lowering your motor to drain water** and any tiny invasive species inside.
