# Supplementary figures and images for: Influencing Aquatic Invasive Species Prevention Behaviors: An Exploration and Experiment with Augmented Reality
Source: Environ Manage. 2025 Oct 1;75(12):3449–60. doi: 10.1007/s00267-025-02283-2 (PMC12575555; doi:10.1007/s00267-025-02283-2)

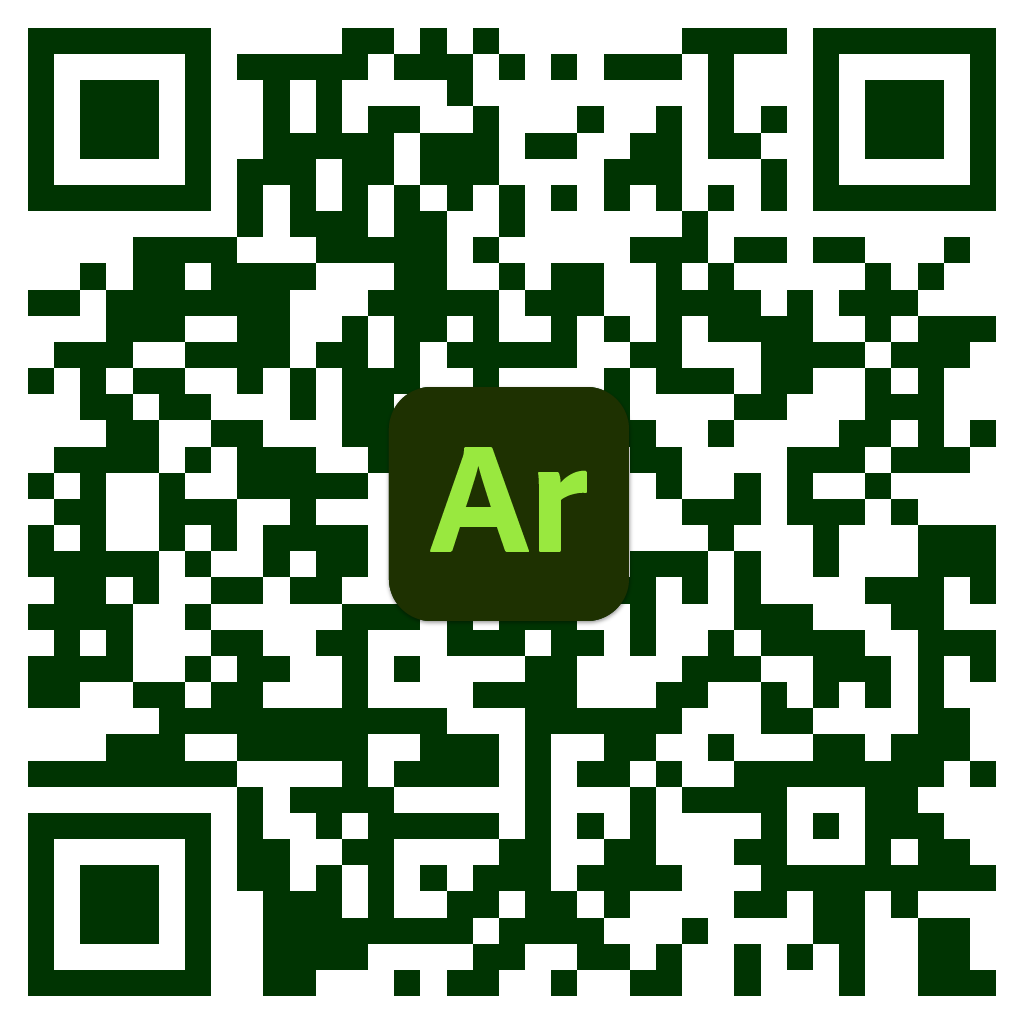

Supplement: Supplementary file 3 — Supplementary information [file 267_2025_2283_MOESM3_ESM.png]

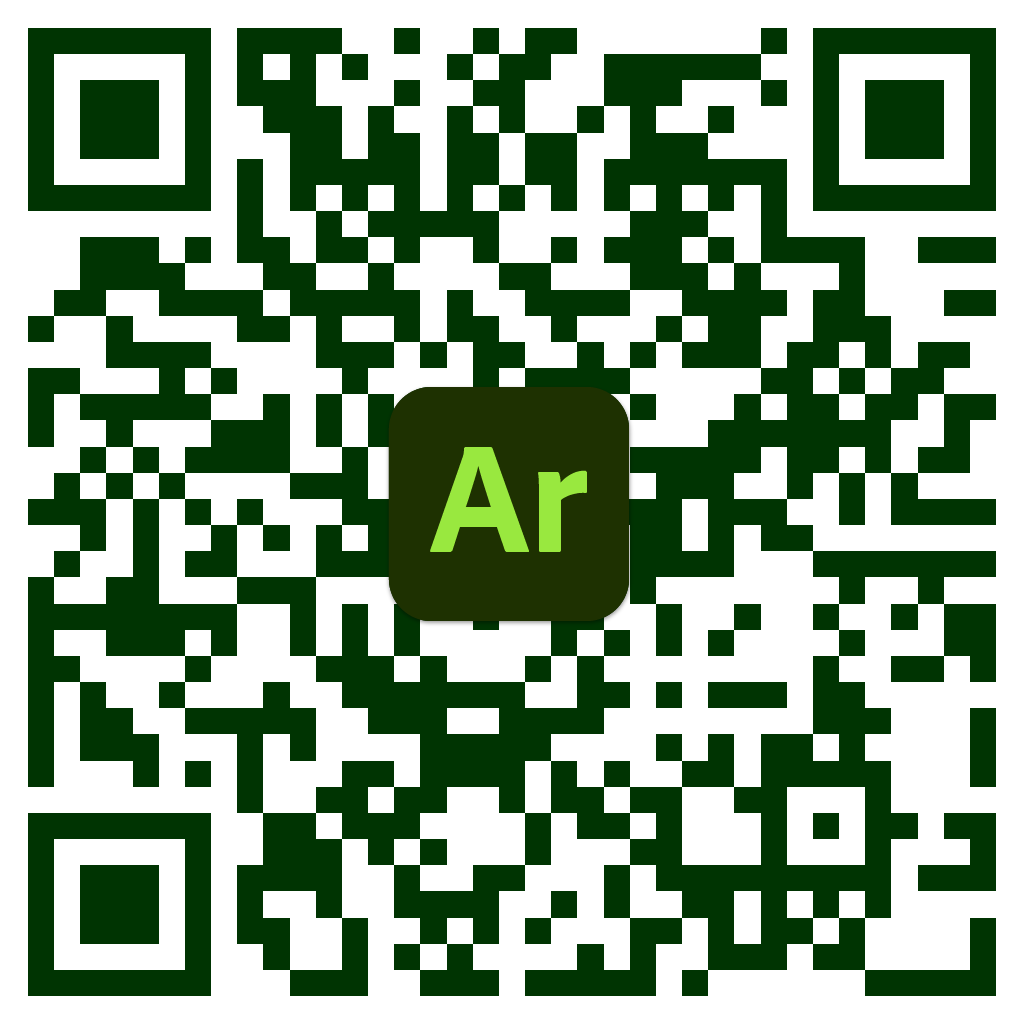

Supplement: Supplementary file 4 — Supplementary information [file 267_2025_2283_MOESM4_ESM.png]

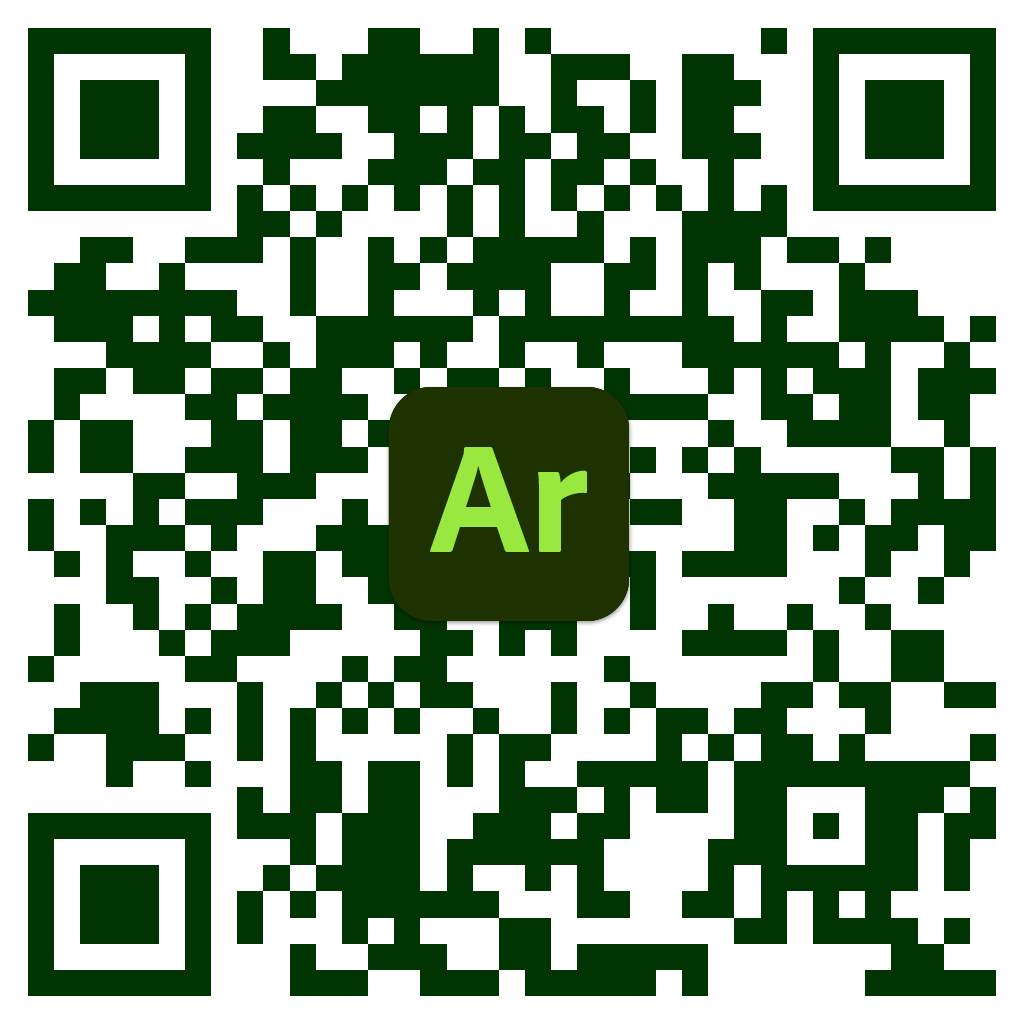

Supplement: Supplementary file 5 — Supplementary information [file 267_2025_2283_MOESM5_ESM.png]
